# Effects of the NEDD8-Activating Enzyme Inhibitor MLN4924 on Lytic Reactivation of Kaposi's Sarcoma-Associated Herpesvirus

**DOI:** 10.1128/JVI.00505-17

**Published:** 2017-09-12

**Authors:** Pey-Jium Chang, Lee-Wen Chen, Li-Yu Chen, Chien-Hui Hung, Ying-Ju Shih, Shie-Shan Wang

**Affiliations:** a Graduate Institute of Clinical Medical Sciences, College of Medicine, Chang-Gung University, Taoyuan, Taiwan; b Department of Nephrology, Chang-Gung Memorial Hospital, Chiayi, Taiwan; c Department of Respiratory Care, Chang-Gung University of Science and Technology, Chiayi, Taiwan; d Department of Pediatric Surgery, Chang-Gung Memorial Hospital, Chiayi, Taiwan; e School of Traditional Chinese Medicine, College of Medicine, Chang-Gung University, Taoyuan, Taiwan; Northwestern University

**Keywords:** KSHV, MLN4924, ORF50, LANA, lytic reactivation, Kaposi's sarcoma-associated herpesvirus, lytic reactivation

## Abstract

The switch of Kaposi's sarcoma-associated herpesvirus (KSHV) from latency to lytic replication is a key event for viral dissemination and pathogenesis. MLN4924, a novel neddylation inhibitor, reportedly causes the onset of KSHV reactivation but impairs later phases of the viral lytic program in infected cells. Thus far, the molecular mechanism involved in the modulation of the KSHV lytic cycle by MLN4924 is not yet fully understood. Here, we confirmed that treatment of different KSHV-infected primary effusion lymphoma (PEL) cell lines with MLN4924 substantially induces viral lytic protein expression. Due to the key role of the virally encoded ORF50 protein in the latent-to-lytic switch, we investigated its transcriptional regulation by MLN4924. We found that MLN4924 activates the ORF50 promoter (ORF50p) in KSHV-positive cells (but not in KSHV-negative cells), and the RBP-Jκ-binding elements within the promoter are critically required for MLN4924 responsiveness. In KSHV-negative cells, reactivation of the ORF50 promoter by MLN4924 requires the presence of the latency-associated nuclear antigen (LANA). Under such a condition, LANA acts as a repressor to block the ORF50p activity, whereas MLN4924 treatment relieves LANA-mediated repression. Importantly, we showed that LANA is a neddylated protein and can be deneddylated by MLN4924. On the other hand, we revealed that MLN4924 exhibits concentration-dependent biphasic effects on 12-*O*-tetradecanoylphorbol-13-acetate (TPA)- or sodium butyrate (SB)-induced viral reactivation in PEL cell lines. In other words, low concentrations of MLN4924 promote activation of TPA- or SB-mediated viral reactivation, whereas high concentrations of MLN4924, conversely, inhibit the progression of TPA- or SB-mediated viral lytic program.

**IMPORTANCE** MLN4924 is a neddylation (NEDD8 modification) inhibitor, which currently acts as an anti-cancer drug in clinical trials. Although MLN4924 has been reported to trigger KSHV reactivation, many aspects regarding the action of MLN4924 in regulating the KSHV lytic cycle are not fully understood. Since the KSHV ORF50 protein is the key regulator of viral lytic reactivation, we focus on its transcriptional regulation by MLN4924. We here show that activation of the ORF50 gene by MLN4924 involves the relief of LANA-mediated transcriptional repression. Importantly, we find that LANA is a neddylated protein. To our knowledge, this is the first report showing that neddylation occurs in viral proteins. Additionally, we provide evidence that different concentrations of MLN4924 have opposite effects on TPA-mediated or SB-mediated KSHV lytic cycle activation. Therefore, in clinical application, we propose that MLN4924 needs to be used with caution in combination therapy to treat KSHV-positive subjects.

## INTRODUCTION

Kaposi's sarcoma-associated herpesvirus (KSHV), also referred to as human herpesvirus 8 (HHV-8), is known as an etiologic agent of Kaposi's sarcoma (KS) and two types of lymphoproliferative diseases, including primary effusion lymphoma (PEL) and multicentric Castleman's disease (1–3). The virus exhibits two distinct phases of its life cycle, latency and lytic replication (4). Latency is characterized by persistence of the viral genome with expression of only a small subset of viral genes. These viral gene products expressed during latency include the latency-associated nuclear antigen (LANA; encoded by open reading frame 73 [ORF73]), viral cyclin (v-cyclin; encoded by ORF72), viral FLIP (v-FLIP; encoded by ORF71), and kaposin (encoded by ORF-K12), as well as 12 microRNAs (5). Once the virus is reactivated from latency and enters lytic replication, viral lytic genes are fully expressed in an orderly fashion, i.e., immediate early, early, and late gene expression, ultimately leading to the production of infectious virions (4). Evidence has shown that multiple viral gene products expressed from both latent and lytic programs have been implicated in the pathogenesis of KSHV-associated diseases (4, 6, 7).

Although the authentic physiological determinants that trigger the transition from latency to lytic replication of KSHV are not fully understood, different chemical, biological, or environmental stimuli, such as 12-*O*-tetradecanoylphorbol-13-acetate (TPA), sodium butyrate (SB), hypoxia, reactive oxygen species, endoplasmic reticulum stress, and proinflammatory cytokines, have been reported to promote viral reactivation in latently infected cells (8–14). During the onset of viral lytic reactivation, a transcriptional activator encoded by open reading frame 50 (ORF50) of the viral genome is expressed at the earliest stage and serves as a key regulator for the progression of viral lytic reactivation (14, 15). In particular, ectopic expression of ORF50 protein alone in latently infected cells is sufficient to drive the entire lytic cascade to completion (16, 17). Numerous viral or cellular transcriptional factors involved in the regulation of ORF50 gene transcription have been characterized, which include LANA, ORF50, RBP-Jκ, KAP1, Nrf2, HIF-1α, AP1, SP1, Oct-1, and C/EBP-α. Among these transcription factors, LANA is known to repress the ORF50 promoter (ORF50p) function through interaction with RBP-Jκ, Nrf2, or the corepressor KAP1 (18–20). In response to stressful stimuli (e.g., hypoxia, TPA, or SB), cellular transcription factors, including HIF-1α, AP1, or SP1, could be activated and act as positive regulators to stimulate ORF50p in infected cells (21–23). In addition to these cellular transcription factors, ORF50 is capable of auto-stimulating its own promoter through its association with RBP-Jκ, Oct-1, or C/EBP-α (24–27). Thus, different internal or external insults that cause dysfunction of these transcription factors in KSHV-infected cells may contribute to the switch between viral latency and lytic replication.

NEDD8 is a ubiquitin-like protein that can be covalently conjugated to target proteins in a manner similar to ubiquitination (28, 29). NEDD8 modification (neddylation) is best known as a posttranslational modification of cullin subunits of cullin-RING ubiquitin ligases (CRLs), the largest category of E3 ubiquitin ligases (30). Conjugation of NEDD8 to the cullin subunit is required for activation of CRLs (31). In addition to cullins, an increasing number of noncullin targets for neddylation have also been reported in recent years (29). Due to the involvement of CRLs in a diverse array of essential cellular processes, alteration of NEDD8 modification in CRLs may potentially affect multiple cellular activities. MLN4924 is a potent neddylation inhibitor that blocks the covalent attachment of NEDD8 to cullins or other target proteins through inhibiting the NEDD8-activating enzyme (32). A growing body of evidence has shown that MLN4924 is a promising anti-cancer drug for a variety of human malignancies (28, 32). Recently, Hughes et al. (33) reported that treatment of KSHV-infected PEL cells with MLN4924 elicits cellular cytotoxicity in a dose-dependent manner. They also noticed that MLN4924 treatment causes induction of viral lytic gene expression, albeit lacking a substantial viral lytic DNA replication, in these treated PEL cells (33). The induction of viral lytic gene expression may be potentially problematic from a therapeutic view because several early lytic gene products possess growth-regulatory or immunomodulatory functions, which are critically involved in the development of KSHV-associated diseases (6, 14). Therefore, it is important to know how MLN4924 triggers the expression of viral lytic genes in infected cells. Furthermore, the combined effects of MLN4924 and other lysis-inducing agents on the progression of the viral lytic program in infected cells also remain obscure.

In this study, we confirmed that MLN4924 indeed triggers the onset of KSHV lytic reactivation in different PEL cell lines. Mechanistically, we showed that MLN4924 induces the ORF50p activity mainly through relieving its transcriptional repression mediated by LANA. Particularly, we found that LANA is a neddylated protein and can be deneddylated by MLN4924 treatment. Moreover, we revealed that different concentrations of MLN4924 display opposite effects on TPA-mediated or SB-mediated lytic reactivation in KSHV-infected cells. Understanding the detailed actions of MLN4924 in the onset or progression of the KSHV lytic program may be helpful in obtaining clues to further elucidate the complicated control mechanisms of the viral lytic cycle.

## RESULTS

### Induction of KSHV lytic gene expression in PEL cells by MLN4924.

**FIG 1:**
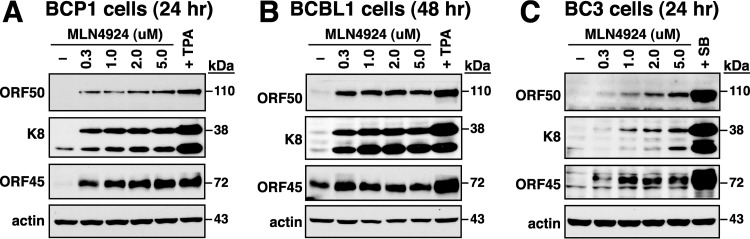
MLN4924 disrupts viral latency in PEL cell lines. Three PEL cell lines, BCP1 (A), BCBL1 (B), and BC3 (C), were treated with different concentrations (0.3, 1.0, 2.0, and 5.0 μM) of MLN4924. In parallel, cells treated with TPA or sodium butyrate (SB) were included as positive controls. At 24 or 48 h posttreatment, the expression of viral lytic proteins, including ORF50, K8, and ORF45, was detected by Western blotting.

To determine whether MLN4924 induces viral lytic gene expression in infected cells, three primary effusion lymphoma (PEL) cell lines, including BCP1, BCBL1, and BC3, were used in the analysis. Since the optimal time points for viral early lytic protein expression in BCP1, BCBL1, and BC3 cells are known to be either 24 or 48 h after treatment with TPA or SB (34), we evaluated the effect of MLN4924 on viral lytic reactivation in these PEL cells at either 24 or 48 h posttreatment. Treatment of BCP1, BCBL1, or BC3 cells with increasing amounts (0.3, 1.0, 2.0, and 5.0 μM) of MLN4924 substantially induced the expression of viral lytic proteins, including ORF50, K8, and ORF45 (Fig. 1A to C). Particularly, in BCP1 and BCBL1 cells, we found that the ability of MLN4924 at high concentrations to induce viral lytic protein expression was similar to that of TPA (Fig. 1A and B). Although the lysis-inducing ability of MLN4924 was lower than that of SB in BC3 cells, MLN4924 treatment promoted viral lytic protein expression in a dose-dependent manner (Fig. 1C). These results indicated that MLN4924 indeed triggers viral reactivation in PEL cells.

### Effects of MLN4924 on the expression of viral and cellular transcriptional regulators.

**FIG 2:**
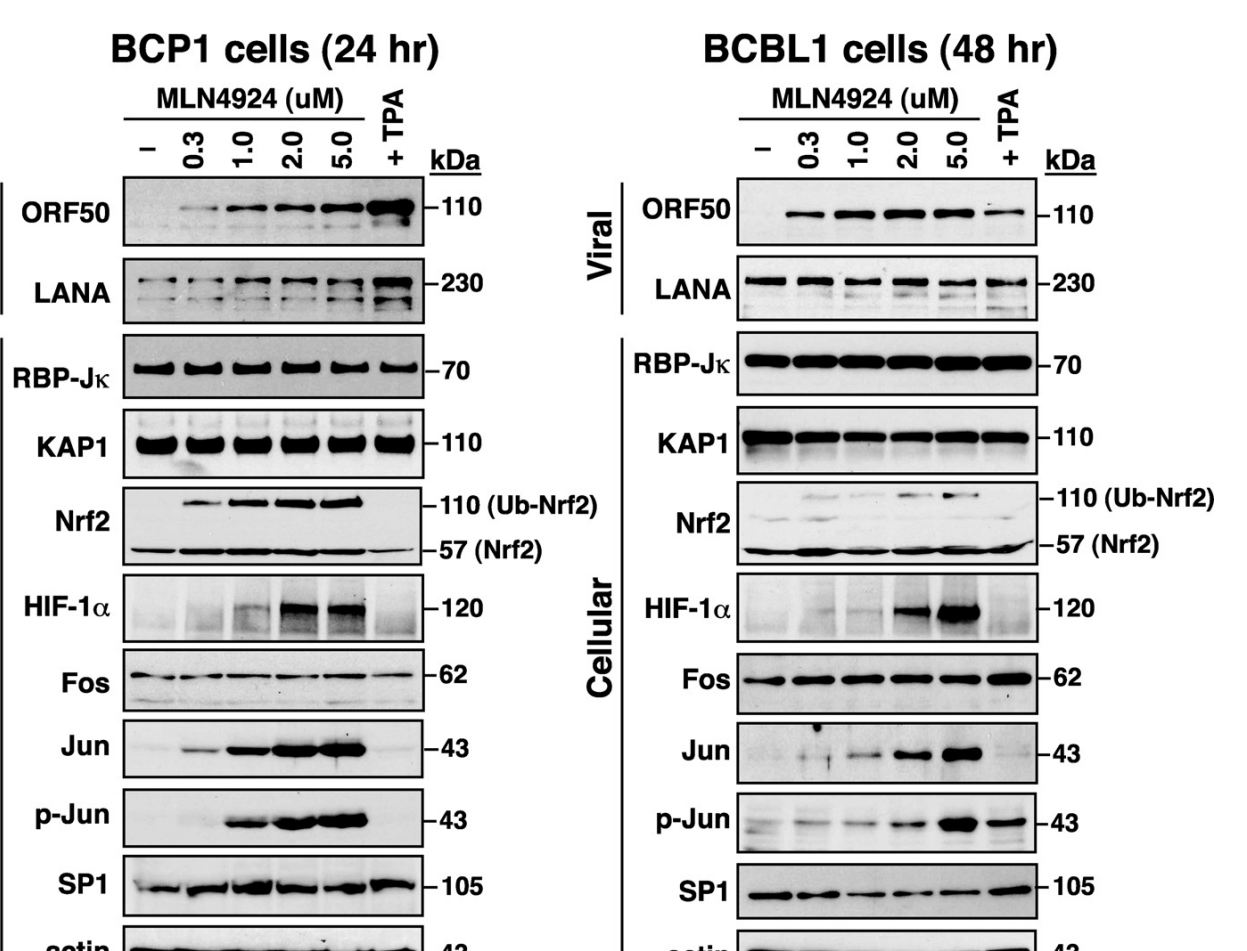
Effects of MLN4924 treatment on the expression of viral and cellular transcriptional regulators in PEL cell lines. Increasing amounts of MLN4924 were used to treat BCP1 and BCBL1 cells for 24 and 48 h, respectively. The expression levels of viral (ORF50 and LANA) and cellular (RBP-Jκ, KAP1, Nrf2, HIF-1α, c-Fos, c-Jun, phosphorylated c-Jun, SP1, and actin) transcriptional regulators were determined by immunoblotting. Ub, ubiquitin.

As shown above, MLN4924 treatment disrupted viral latency and led to viral lytic gene expression in PEL cells. This observation raised the possibility that treatment of these PEL cells with MLN4924 might cause deregulation of specific viral or cellular transcriptional factors that are required for ORF50 gene expression. To test this possibility, we examined the protein expression of LANA, RBP-Jκ, KAP1, Nrf2, HIF-1α, Fos, Jun, and SP1 in BCP1 and BCBL1 cells after MLN4924 treatment (Fig. 2). Western blot analysis revealed that the steady-state level of LANA was only slightly increased in BCP1 cells, but not in BCBL1 cells, after exposure to increasing amounts of MLN4924. Similarly, MLN4924 treatment also did not significantly influence the protein levels of RBP-Jκ, KAP1, Fos, and SP1 in BCP1 and BCBL1 cells. However, we found that Nrf2 (especially ubiquitinated Nrf2), HIF-1α, Jun, and phosphorylated Jun (p-Jun) in BCP1 and BCBL1 cells were remarkably increased by MLN4924. These results suggested that the deregulated Nrf2, HIF-1α, Jun, or p-Jun resulting from MLN4924 exposure might play critical roles in the induction of the ORF50 gene in BCP1 and BCBL1 cells.

### Mapping of MLN4924-responsive elements in the ORF50 promoter.

**FIG 3:**
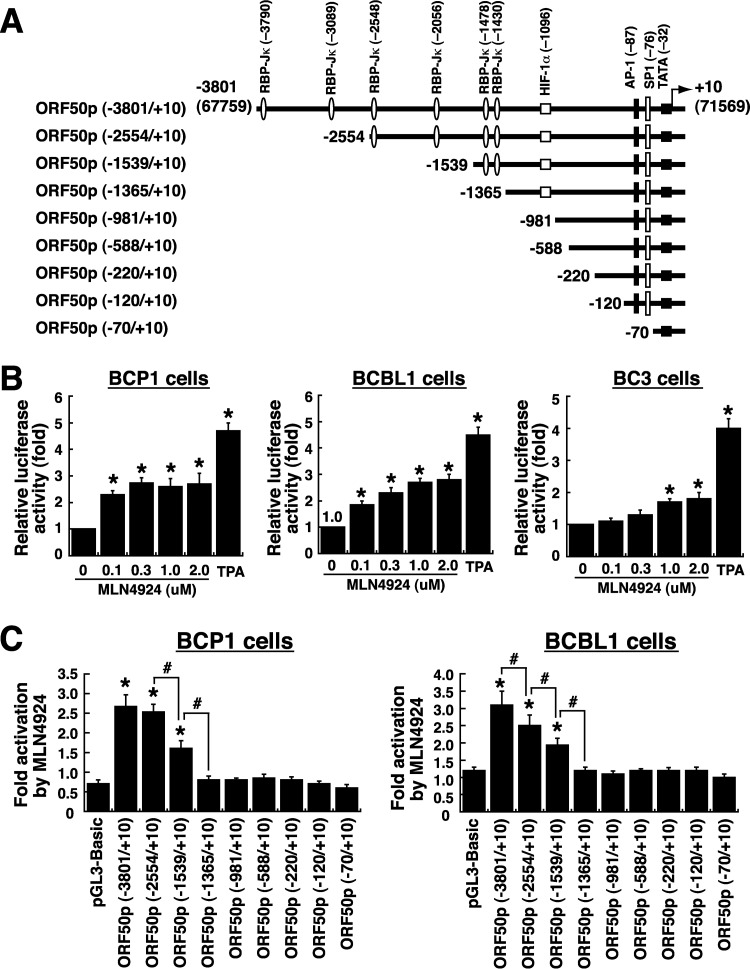
Mapping of the MLN4924-responsive element in the ORF50 promoter. (A) Schematic diagram of the full-length and deleted ORF50 promoters in reporter constructs. Several known binding sites for transcription factors in the ORF50 promoter are indicated. (B) Transcriptional activation of the full-length ORF50 promoter (−3801/+10) by MLN4924 in PEL cells. The pORF50p(−3801/+10)/luc reporter construct was transfected into different PEL cell lines (BCP1, BCBL1, and BC3), and the transfected cells were left untreated or treated with different concentrations of MLN4924 for 24 h. The relative luciferase activity of the reporter construct in untreated or MLN4924-treated cells was measured as described in Materials and Methods. Asterisks indicate significant differences in results versus those with the untreated control (*P* < 0.05). (C) Responses of the ORF50p deletion constructs to MLN4924. BCP1 and BCBL1 cells were transfected with indicated reporter plasmids, and the transfected cells were left untreated or treated with MLN4924 (0.3 μM). Activation of each deleted ORF50p reporter construct by MLN4924 was determined at 24 h after MLN4924 treatment. *, *P* < 0.05, for results compared to those with pGL3-Basic; #, *P* < 0.05, for results compared to those with the indicated controls.

We simultaneously examined whether MLN4924 transcriptionally regulates the ORF50 promoter (ORF50p) in PEL cells. To do this experiment, a luciferase (luc) reporter plasmid pORF50p(−3801/+10)/luc that contains a 3.8-kb ORF50p region (Fig. 3A) was transfected into BCP1, BCBL1, or BC3 cells. The transfected cells were then treated with various concentrations of MLN4924 from 0.1 μM to 2.0 μM for 24 h. In all tested PEL cells, we found that the ORF50p activity was gradually enhanced by increasing amounts of MLN4924 (Fig. 3B). In agreement with the Western blot analysis shown in Fig. 1, the increased ORF50p activity caused by MLN4924 in BCP1 and BCBL1 cells was higher than that in BC3 cells (1.9- to 2.8-fold increase versus 1.1- to 1.8-fold increase). Notably, we failed to detect activation of the ORF50p reporter construct by MLN4924 in KSHV-negative cell lines (see below).

To map the MLN4924-responsive element in the ORF50 promoter, a series of ORF50p deletion constructs were generated (Fig. 3A). The resultant reporter plasmids were individually transfected into BCP1 or BCBL1 cells, and the transfected cells were left untreated or treated with 0.3 μM MLN4924 (Fig. 3C). When we deleted the ORF50p region from −3801 to −1365, we found that this deleted ORF50p reporter construct, pORF50p(−1365/+10), completely lost its response to MLN4924 in both BCP1 and BCBL1 cells (Fig. 3C). As noted, there are six RBP-Jκ-binding sites located in this promoter region from −3801 to −1365, suggesting that these RBP-Jκ elements could have important roles in the induction of ORF50p transcription by MLN4924. Surprisingly, although MLN4924 treatment led to an increase in protein levels of HIF-1α, Jun, and p-Jun in BCP1 and BCBL1 cells (Fig. 2), the pORF50p(−1365/+10) reporter construct that contains both HIF-1α- and AP1-binding sites could not produce this response to MLN4924 (Fig. 3C).

**FIG 4:**
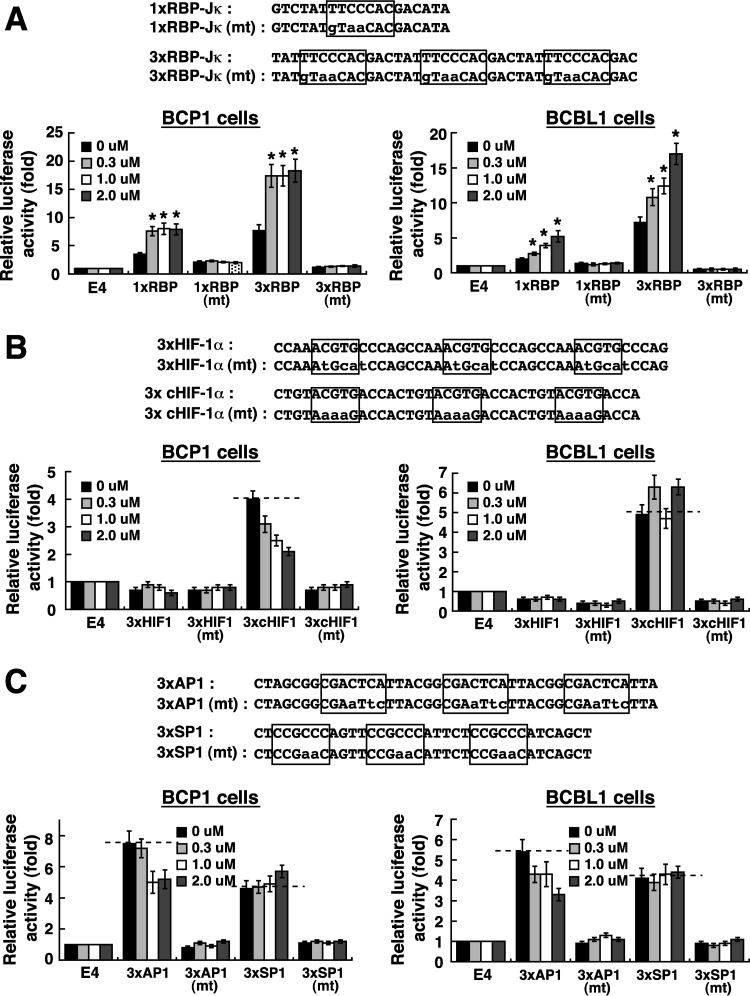
The RBP-Jκ-binding motifs in the ORF50 promoter critically confer MLN4924 responsiveness. (A) Responses of 1×RBP-Jκ- and 3×RBP-Jκ-containing reporter constructs to MLN4924. One or three copies of a RBP-Jκ element or its mutant element (mt) were constructed into pE4luc (E4). The indicated reporter plasmids were individually transfected into BCP1 and BCBL1 cells, and the relative reporter activation by MLN4924 (0.3, 1.0, and 2.0 μM) was measured at 24 h posttreatment. Asterisks indicate significant difference in results versus those with the untreated control (*P* < 0.05). (B) MLN4924 responsiveness of the reporter plasmids containing an HIF-1α-binding element from the ORF50 promoter or a consensus HIF-1α-responsive element (cHIF-1α). 3 × HIF-1α, three copies of viral HIF-1α-binding element from the ORF50 promoter; 3×cHIF-1α, three copies of a consensus HIF-1α response element. (C) Responses of AP1- and SP1-containing reporter constructs to MLN4924. 3×AP1, three copies of an AP1-binding element from the ORF50 promoter; 3×SP1, three copies of an SP1-binding element from the ORF50 promoter. The dashed horizontal lines on the graphs indicate that the reporter activation was maintained at the uninduced level.

To further confirm the importance of individual binding sites of transcription factors within the ORF50 promoter in response to MLN4924, three tandem copies of the RBP-Jκ-, HIF-1α-, AP1- or SP1-binding element (3×RBP-Jκ, 3×HIF-1α, 3×AP1, or 3×SP1, respectively) were inserted into pE4luc, a reporter plasmid with a minimal adenovirus E4 promoter. In parallel, mutant reporter constructs with point mutations in each binding element were also generated (Fig. 4). Generally, the constructed reporter plasmids that encompass wild-type binding elements produced higher basal levels of luciferase activity in cells than their corresponding mutant plasmids or the control vector pE4luc (Fig. 4). When these reporter plasmids were analyzed for their MLN4924 responsiveness in BCP1 or BCBL1 cells, we found that MLN4924 activated only the 3×RBP-Jκ-containing reporter construct but not the reporter constructs that encompass its corresponding mutated element (Fig. 4A) or the HIF-1α-, AP1-, or SP1-binding element (Fig. 4B and C). Particularly, one single copy of the RBP-Jκ-binding element was sufficient to produce the response to MLN4924 (Fig. 4A, 1×RBP-Jκ). Since the cloned HIF-1α-binding element from the ORF50 promoter did not produce the response to MLN4924 in PEL cells (Fig. 4B, 3×HIF-1α), we additionally tested a consensus HIF-1α response element (cHIF-1α) for its MLN4924 responsiveness (Fig. 4B). Similarly, MLN4924 treatment still could not mediate activation of the cHIF-1α-containing reporter construct (Fig. 4B, cHIF-1α). Our results therefore indicated that the RBP-Jκ-binding motifs in the ORF50 promoter are the MLN4924-responsive element in PEL cells.

### Involvement of LANA in MLN4924-mediated ORF50p activation.

**FIG 5:**
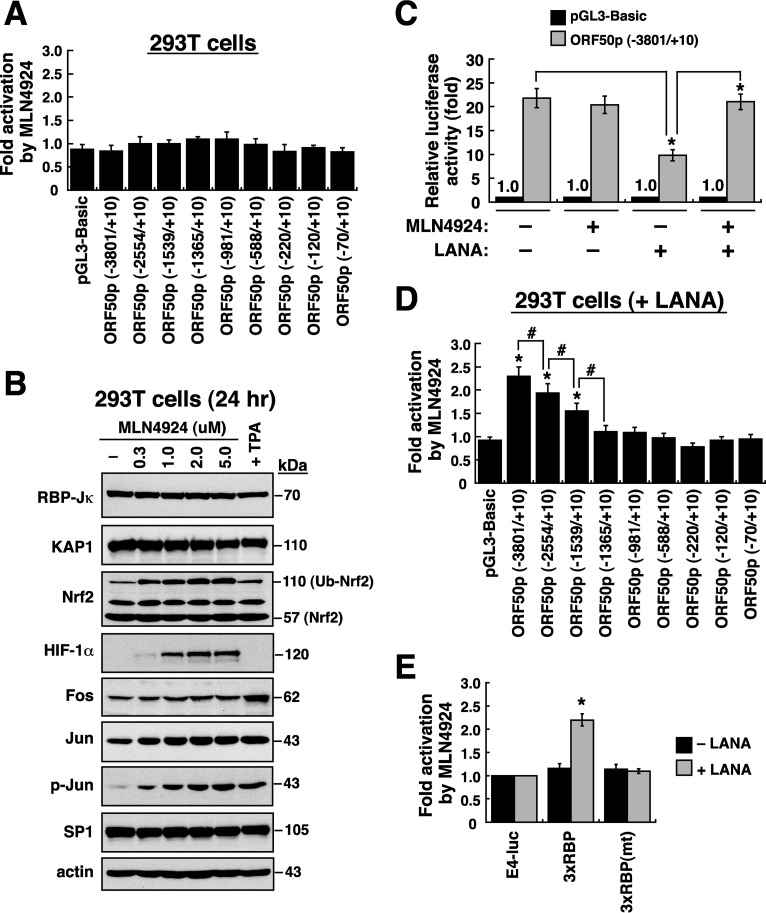
MLN4924 relieves LANA-mediated repression of the ORF50 promoter in 293T cells. (A) MLN4924 responsiveness of the ORF50p-driven reporter constructs in 293T cells. 293T cells were transfected with the indicated reporter plasmids and then left untreated or treated with MLN4924 (1.0 μM) for 24 h. The fold activation of these reporter constructs by MLN4924 was determined as the luciferase activity in the presence of MLN4924 divided by luciferase activity in the absence of MLN4924. (B) Western blot analysis of specific cellular transcription factors expressed in 293T cells after treatment with MLN4924 (0.3, 1.0, 2.0, and 5.0 μM) for 24 h. (C) Involvement of LANA in the MLN4924-mediated ORF50p activation in 293T cells. The pORF50p(−3801/+10)/luc reporter construct or the pGL3-Basic reporter vector was cotransfected with the empty vector or the LANA expression plasmid into 293T cells. The transfected cells were left untreated or treated with MLN4924 (1.0 μM) for 24 h. The luciferase activity of pORF50p(−3801/+10)/luc under different treatment conditions was measured relative to the activity of the empty reporter vector pGL3-Basic. *, *P* < 0.05, for results compared to those with the indicated controls. (D) MLN4924 responsiveness of the ORF50p reporter constructs in LANA-transfected 293T cells. The indicated reporter constructs were individually cotransfected with the LANA expression plasmid into 293T cells, and their MLN4924 responsiveness was determined in these transfected cells after treatment with MLN4924 (1.0 μM) for 24 h. *, *P* < 0.05, for results compared to those with pGL3-Basic; #, *P* < 0.05, for results compared to those with the indicated controls. (E) Activation of the 3×RBP-Jκ-containing reporter construct by MLN4924 in LANA-transfected 293T cells. *, *P* < 0.05, for results compared to those with pE4luc.

Although MLN4924 substantially induced the ORF50 promoter in PEL cells (Fig. 3), we detected no activation of the ORF50p-directed reporter constructs by MLN4924 in 293T or HKB5/B5 cells, two KSHV-negative cell lines (Fig. 5A and data not shown). Two possibilities could be proposed to explain these results. One possibility is that 293T or HKB5/B5 cells could not respond to MLN4924 treatment; the other possibility is that activation of the ORF50 promoter by MLN4924 might require the expression of specific viral factors. The former hypothesis seemed unlikely because MLN4924 treatment normally increased the protein levels of Nrf2, HIF-1α, Jun, and p-Jun in 293T and HKB5/B5 cells (Fig. 5B and data not shown). In connection with the latter hypothesis, two viral proteins including LANA and ORF50 were considered because both viral proteins are known to antagonistically regulate the ORF50 promoter via RBP-Jκ-binding sites (18, 25, 27). To test the potential involvement of ORF50 and LANA in MLN4924-mediated ORF50p activation, we cotransfected the reporter construct pORF50p(−3801/+10)/luc with the expression plasmid for LANA or ORF50 into 293T cells. Although ORF50 expression indeed increased the reporter activity of pORF50p(−3801/+10)/luc in 293T cells up to 6-fold, MLN4924 treatment could not further augment the already elevated reporter activity (data not shown). In contrast, we found that LANA expression reduced the basal reporter activity of pORF50p(−3801/+10)/luc by 55% in 293T cells; however, treatment with MLN4924 under such a condition restored ORF50p activity (Fig. 5C). To further confirm the importance of LANA in MLN4924-mediated ORF50p activation, different ORF50p deletion reporter constructs were tested for their MLN4924 responsiveness in 293T cells by cotransfection with the LANA expression plasmid (Fig. 5D). We found that activation patterns of these ORF50p deletion constructs by MLN4924 in the LANA-transfected 293T cells were very similar to those observed in PEL cells (compare Fig. 3C and 5D). Moreover, we also demonstrated that LANA was required for activation of the 3×RBP-Jκ-containing reporter construct by MLN4924 in 293T cells (Fig. 5E). Taken together, our results strongly suggested that activation of the ORF50 promoter by MLN4924 involves the relief of the LANA/RBP-Jκ-mediated transcriptional repression.

### Neddylation of LANA in cells.

**FIG 6:**
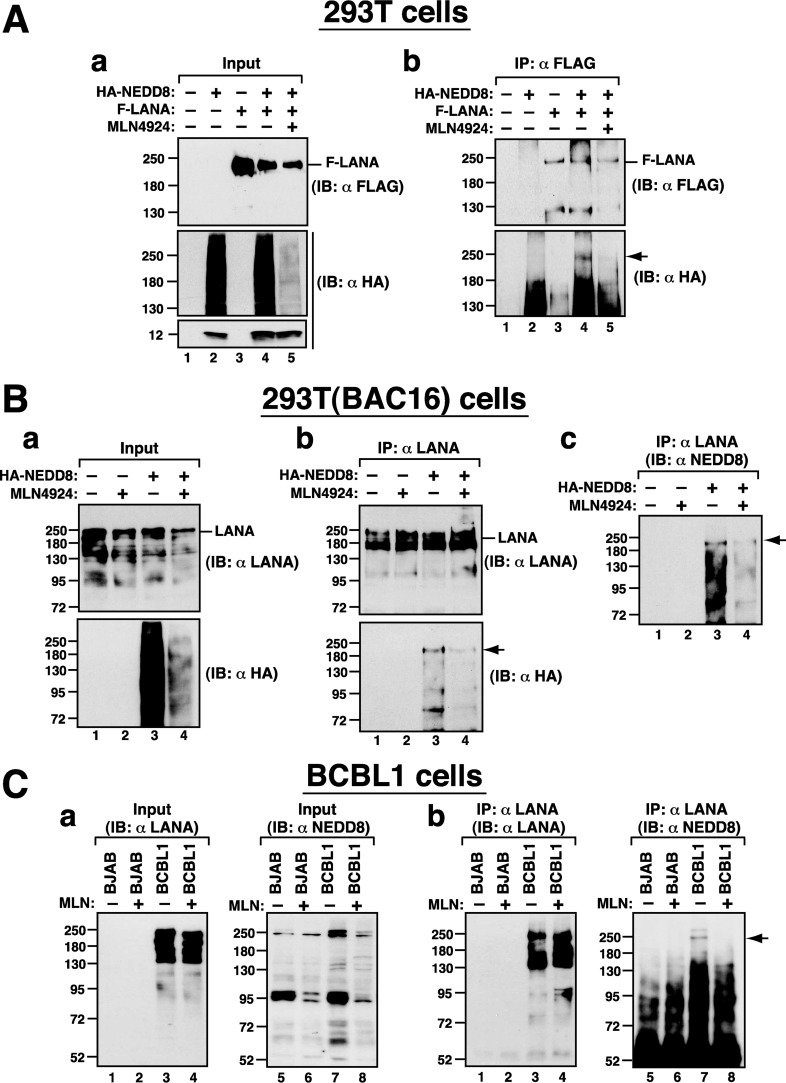
LANA is naturally modified with NEDD8 in cells. (A) Neddylation of LANA in 293T cells. 293T cells were transfected with the indicated plasmids expressing HA-NEDD8 or F-LANA and left untreated or treated with MLN4924 (2.0 μM) for 24 h. Cell samples were then subjected to immunoprecipitation (IP) and immunoblot (IB) analysis. (B) Neddylation of LANA in 293T(BAC16) cells. 293T(BAC16) cells were transfected with the expression plasmid for HA-NEDD8 and cultured in medium with or without MLN4924 (2.0 μM) for 24 h. Cell lysates were immunoprecipitated using anti-LANA antibody, and the resultant immunoprecipitates were analyzed by immunoblotting using antibodies against LANA, HA, and NEDD8. (C) Neddylation of LANA in BCBL1 cells. BJAB and BCBL1 cells were left untreated or treated with MLN4924 (2.0 μM) for 24 h. After cell lysates were immunoprecipitated with anti-LANA antibody, the immunoprecipitated proteins were probed with anti-LANA and anti-NEDD8 antibodies. Arrows indicate the positions of the neddylated LANA.

Since the levels of LANA and RBP-Jκ proteins were not significantly changed after treatment with MLN4924 in PEL cells, we therefore tested whether LANA could be a neddylated protein and whether MLN4924 treatment could inhibit LANA neddylation. Accordingly, 293T cells were cotransfected with the plasmids that express FLAG-tagged LANA (F-LANA) and/or hemagglutinin (HA)-tagged NEDD8 (HA-NEDD8), and the transfected cells were left untreated or treated with MLN4924 (2 μM) for 24 h. Notably, although MLN4924 treatment greatly reduced conjugation of HA-NEDD8 to target proteins in cells (Fig. 6A, graph a, compare lanes 4 and 5), we did not find more free HA-NEDD8 (12 kDa) in MLN4924-treated cells than in untreated cells (Fig. 6A, graph a, compare lanes 4 and 5). The explanation of this outcome could be that MLN4924 treatment profoundly impaired the expression of HA-NEDD8 from the transfected expression vector or promoted the degradation of free HA-NEDD8 in cells. Cell lysates were immunoprecipitated with anti-FLAG antibody, and then the immunoprecipitates were immunoblotted with anti-FLAG or anti-HA antibody (Fig. 6A, graph b). Similar amounts of F-LANA were immunoprecipitated in all F-LANA-transfected cell samples, irrespective of the presence or absence of HA-NEDD8 or MLN4924 (Fig. 6A, graph b, top panel). Immunoblotting analysis using anti-HA antibody showed that a band corresponding to the full-length F-LANA (∼230 kDa) was detected only in the immunoprecipitate from the F-LANA/HA-NEDD8-cotransfected, MLN4924-untreated cell sample (Fig. 6A, graph b, lane 4). However, such a protein band could not be observed in the immunoprecipitates from the F-LANA-transfected cell sample (Fig. 6A, graph b, lane 3) and from the F-LANA/HA-NEDD8-cotransfected, MLN4924-treated cell sample (Fig. 6A, graph b, lane 5). These results indicated that LANA could be a neddylated protein.

To further confirm our findings, 293T(BAC16) cells (where BAC16 is bacterial artificial chromosome 16 carrying the KSHV genome) that were transfected with the HA-NEDD8 expression plasmid were subjected to immunoprecipitation with anti-LANA antibody (Fig. 6B). The resultant immunoprecipitates were subsequently analyzed by immunoblotting using antibodies against LANA, HA, and NEDD8. We consistently found that endogenous LANA in 293T(BAC16) cells was neddylated, as detected in immunoblot analysis using anti-HA or anti-NEDD8 antibody (Fig. 6B, graphs b and c, lanes 3). Importantly, treatment of the transfected 293T(BAC16) cells with MLN4924 markedly reduced LANA neddylation (Fig. 6B, graphs b and c, lanes 4). Moreover, LANA neddylation was also examined in BCBL1 cells. After immunoprecipitation using anti-LANA antibody, we found that endogenous LANA in BCBL1 cells was also neddylated, as demonstrated by immunoblotting using anti-NEDD8 antibody (Fig. 6C, graph b, lane 7). Consistently, MLN4924 treatment resulted in a decrease of LANA neddylation in BCBL1 cells (Fig. 6C, graph b, lane 8).

### Effects of MLN4924 on the progression of KSHV lytic reactivation.

**FIG 7:**
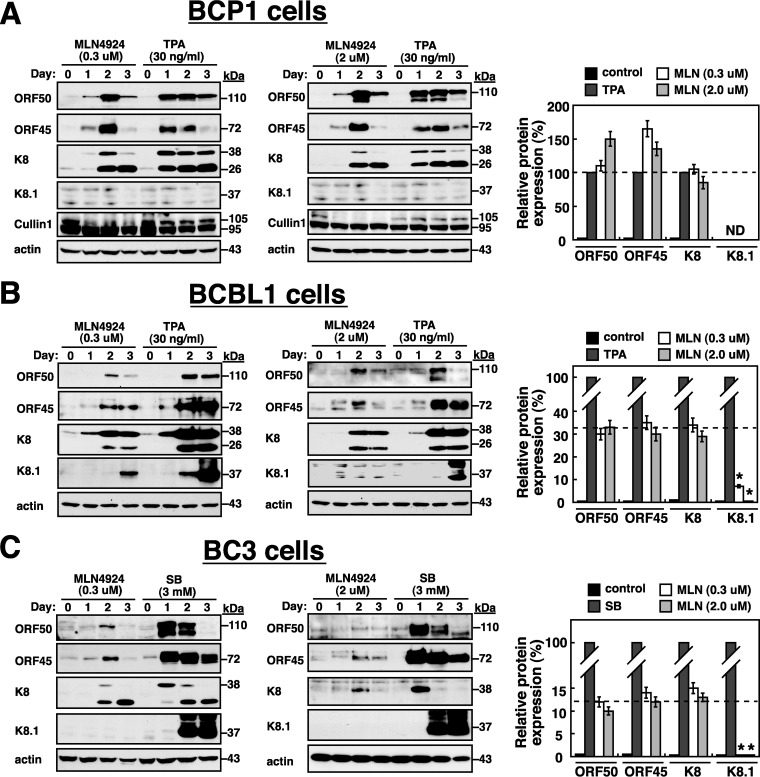
Treatment of PEL cells with MLN4924 triggers the expression of early lytic proteins but not the late lytic protein K8.1. MLN4924 (MLN) at 0.3 μM or 2.0 μM was used to treat BCP-1 (A), BCBL1 (B), and BC3 (C) cells. At different time points (1, 2, and 3 days) after treatment, the expression levels of viral lytic proteins in these PEL cell lines were determined by Western blotting. In parallel, cell samples treated with TPA or SB were also included as positive controls. Bar graphs show densitometry quantification of the maximal expression level of each viral lytic protein induced by MLN4924 (0.3 or 2.0 μM) relative to that induced by TPA or SB. As noted, K8.1 protein expression was undetectable in BCP1 cells even after treatment with TPA. The dashed horizontal lines on the graphs represent the average expression levels of ORF50, ORF45, and K8 induced by MLN4924 in PEL cells. Asterisks indicate significant difference between K8.1 induction and induction of other viral lytic proteins after MLN4924 treatment (*P* < 0.05). ND, not detected.

To determine whether MLN4924 was sufficient to drive the entire KSHV lytic cycle to completion in PEL cells, the expression levels of viral immediate early, early, and late proteins were examined in PEL cell lines after treatment with MLN4924 at either 0.3 μM (low concentration) or 2 μM (high concentration). In parallel, PEL cells that were treated with TPA or SB were included as positive controls. The immediate early and early lytic proteins, including ORF50, ORF45, and K8, were evidently detected in all tested PEL cell lines after treatment with either 0.3 μM or 2 μM MLN4924 (Fig. 7A to C). Compared to results of TPA treatment in BCP1 cells or SB treatment in BC3 cells, MLN4924 treatment in these cells resulted in a delayed induction of ORF50, ORF45, and K8, with the maximal protein level at 48 h posttreatment (Fig. 7A and C). Although MLN4924 substantially induced ORF50, ORF45, and K8 in all tested PEL cells, the expression level of the late lytic protein K8.1 was very low or undetectable in these MLN4924-treated cells (Fig. 7A to C). For example, in BCBL1 cells, the maximal induction of ORF50, ORF45, and K8 by MLN4924 at 0.3 μM or 2 μM was up to 30 to 35% of the maximal levels of their corresponding proteins induced by TPA. However, the maximal level of K8.1 induced by MLN4924 in BCBL1 cells was less than 5% of the maximal K8.1 induction by TPA (Fig. 7B). Similar effects of MLN4924 on the induction of these viral lytic proteins were also seen in BC3 cells (Fig. 7C). These results indicated that MLN4924 has profound effects on the progression of the KSHV lytic program. During the course of experiments, we unexpectedly found that even treatment of BCP1 cells with TPA could not induce K8.1 expression (Fig. 7A), suggesting that certain viral or cellular factors essential for the progression of the viral lytic cycle cascade might be defective in BCP1 cells.

**FIG 8:**
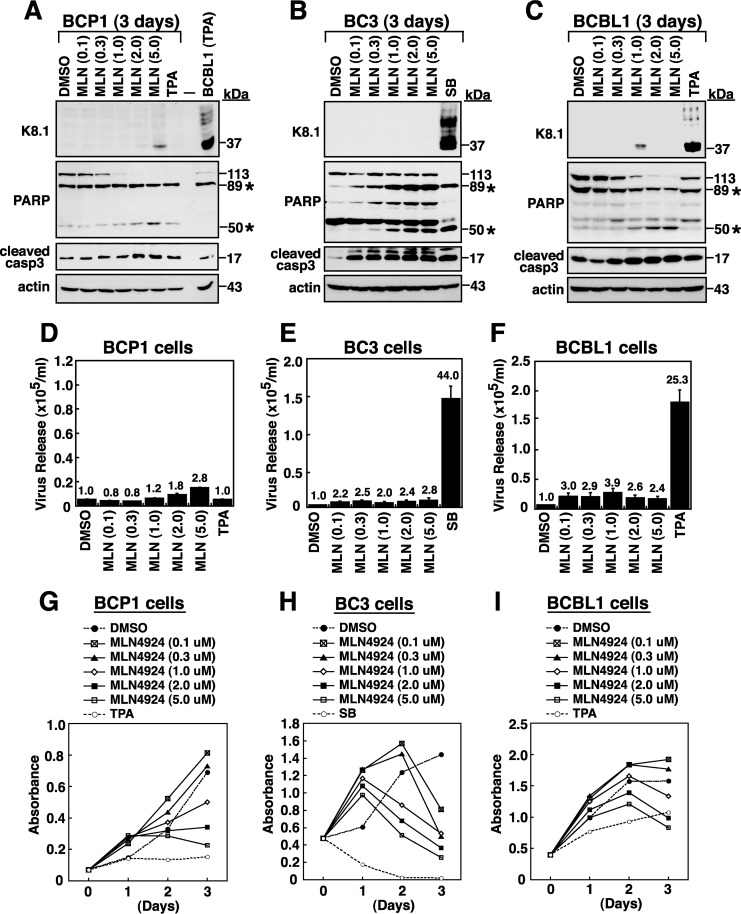
The blockage of KSHV lytic progression by MLN4924 is not due to cellular cytotoxicity. (A to C) Western blot detection of K8.1 as well as cleaved PARP and caspase-3 in BCP1, BC3, and BCBL1 cells that were treated with various concentrations of MLN4924 (0.1, 0.3, 1, 2, or 5 μM) for 3 days. In the experiments, PEL cells treated with TPA or SB served as controls. As noted, there are two cleaved PARP fragments (89 and 50 kDa; asterisks) produced in treated cells. (D to F) Quantitative analysis of virus particles from culture supernatants of BCP1, BC3, and BCBL1 cells after treatment with MLN4924, TPA, or SB for 3 days. (G to I) Effects of different concentrations of MLN4924 on cell proliferation of BCP1, BC3, and BCBL1 cells. After PEL cells were left untreated or treated with MLN4924 at the indicated concentrations or with TPA or SB, cell proliferation was measured by XTT assay. DMSO, dimethyl sulfoxide.

To further verify the action of MLN4924 in the progression of the KSHV lytic cycle, the expression of K8.1 and the release of virus particles were evaluated in PEL cells after treatment with different concentrations of MLN4924 (0.1 μM to 5.0 μM) for 3 days (Fig. 8). Compared to TPA or SB treatment in PEL cells, MLN4924 treatment at either concentration did not cause a significant induction of K8.1 expression (Fig. 8A to C). Similarly, the release of virus particles from these MLN4924-treated PEL cells was also detected at the background level (Fig. 8D to F). All of these experiments supported the idea that MLN4924 treatment triggers the onset of viral reactivation but impairs the later stages of the viral lytic cycle. To determine whether the inefficient induction of K8.1 expression and viral particle release were due to rapid cell death caused by MLN4924, the proteolytic cleavage of both poly(ADP-ribose) polymerase (PARP) and caspase-3 as well as cell viability was assessed in these PEL cells. According to Western blot analysis of the cleavage of both PARP and caspase-3 (Fig. 8A to C) and the cell viability assay (Fig. 8G to I), we found that MLN4924 at all tested concentrations (0.1 μM to 5.0 μM) in PEL cells generally displayed lower toxicity than TPA or SB (Fig. 8G to I). Notably, in contrast to results in untreated control cells, we found that treatment of these PEL cell lines with low concentrations (0.1 or 0.3 μM) of MLN4924 significantly elicited a transient increase in cell proliferation over a period of 1 to 2 days (Fig. 8G to I).

### Concentration-dependent biphasic effects of MLN4924 on TPA-mediated or SB-mediated KSHV reactivation.

**FIG 9:**
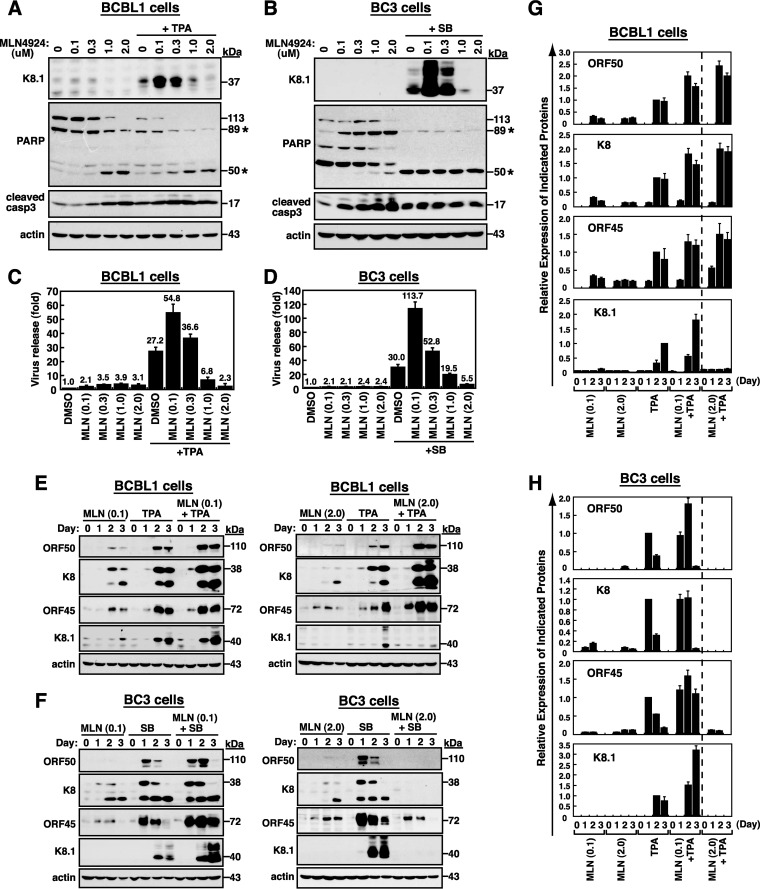
MLN4924 exhibits concentration-dependent biphasic actions in viral lytic-cycle activation in TPA-treated BCBL1 cells and in SB-treated BC3 cells. (A and B) BCBL1 and BC3 cells were treated with TPA and SB, respectively, in combination with various concentrations (0, 0.1, 0.3, 1, and 2 μM) of MLN4924. After 3 days of treatment, treated samples were analyzed for K8.1 expression and both cleaved PARP and caspase-3. As noted, there are two cleaved PARP fragments (89 and 50 kDa; asterisks) detected in treated cells. (C and D) The amounts of virus particles released from the above treated cell samples were measured by quantitative PCR. (E) Kinetics of viral lytic protein expression in BCBL1 cells that were treated with MLN4924 (0.1 or 2.0 μM), TPA, or the combination of MLN4924 (0.1 or 2.0 μM) and TPA. The expression levels of viral lytic proteins were examined by Western blotting using the indicated antibodies. (F) Western blot analysis of viral lytic protein expression in BC3 cells that were treated with MLN4924 (0.1 or 2.0 μM), SB, or the combination of MLN4924 (0.1 or 2.0 μM) and SB. (G and H) Densitometry quantification of viral lytic protein expression under different treatment conditions in BCBL1 cells and in BC3 cells. The dashed vertical lines on the graphs are used to separate the expression patterns of viral lytic proteins induced by 2.0 μM MLN4924 along with TPA or SB in BCBL1 cells or in BC3 cells from those induced by other treatment combinations.

As described above, MLN4924 alone initiated viral lytic gene expression but impaired the later lytic progression. To investigate whether MLN4924 antagonistically influences TPA- or SB-mediated viral reactivation, we treated BCBL1 and BC3 cells with TPA and SB, respectively, along with different concentrations (0.1, 0.3, 1.0, or 2.0 μM) of MLN4924 for 3 days. In the experiments, we found that the increasing concentrations of MLN4924 partially enhanced TPA- or SB-mediated cell apoptosis in BCBL1 and BC3 cells, as detected by cleavage of both PARP and caspase-3 (Fig. 9A and B). When K8.1 expression was examined in these treated cells, we found that low concentrations (0.1 and 0.3 μM) of MLN4924 substantially enhanced the levels of K8.1 in TPA-treated BCBL1 cells and in SB-treated BC3 cells (Fig. 9A and B). However, higher concentrations (1.0 and 2.0 μM) of MLN4924 conversely repressed K8.1 expression in TPA-treated BCBL1 cells and in SB-treated BC3 cells (Fig. 9A and B). Similar concentration-dependent biphasic actions of MLN4924 were also observed when the release of virus particles was measured from cultured BCBL1 cells that were treated with TPA and MLN4924 and from BC3 cells that were treated with SB and MLN4924 (Fig. 9C and D). To further examine the biphasic regulatory mechanisms of MLN4924 in TPA- or SB-mediated KSHV reactivation, time course experiments were performed to analyze the expression of viral lytic proteins in BCBL1 cells or BC3 cells. As expected, treatment of BCBL1 cells or BC3 cells with MLN4924 alone at 0.1 μM (low concentration) or 2.0 μM (high concentration) elicited only a mild increase in viral lytic protein expression compared to levels induced by TPA or SB (Fig. 9E and F). When BCBL1 cells were treated with the combination of 0.1 μM MLN4924 and TPA, all tested viral lytic proteins (including ORF50, K8, ORF45, and K8.1) were expressed at higher levels than those in cells treated with TPA alone (Fig. 9E, left panel, and G). Although treatment of BCBL1 cells with the combination of 2.0 μM MLN4924 and TPA also promoted higher levels of ORF50, K8, and ORF45 than those in TPA-treated BCBL1 cells, we found that K8.1 expression was substantially lower in MLN4924 (2.0 μM)/TPA-treated cells than in TPA-treated cells (Fig. 9E, right panel, and G). On the other hand, in BC3 cells, the combined treatment of SB and 0.1 μM MLN4924 maintained lytic protein expression at higher levels than those in cells treated with SB alone during lytic reactivation (Fig. 9F, left panel, and H). Unlike what we observed in BCBL1 cells, the combined treatment of 2.0 μM MLN4924 and SB in BC3 cells caused nearly complete loss of viral lytic protein induction (Fig. 9F, right panel, and H). Based on these results, we concluded that different concentrations of MLN4924 exhibit opposite effects on TPA-mediated or SB-mediated lytic replication in PEL cell lines. Moreover, MLN4924 may use distinct regulatory mechanisms to modulate viral reactivation in TPA-treated and SB-treated PEL cells.

## DISCUSSION

In this report, we investigate the molecular mechanisms of action of MLN4924 in the onset and progression of KSHV reactivation in PEL cells. There are five major conclusions in the study. First, we confirm that MLN4924 is capable of inducing viral lytic gene expression in different PEL cell lines. Second, we find that MLN4924 activates ORF50 gene transcription in KSHV-infected PEL cells and that the RBP-Jκ-binding sites within the ORF50 promoter are the MLN4924-responsive element. Third, we show that LANA is involved in MLN4924-mediated ORF50p activation. Mechanistic studies reveal that MLN4924 functions to relieve LANA-dependent repression of ORF50p transcription. Fourth, we reveal that LANA is a NEDD8-modified protein. To the best of our knowledge, this is the first report describing NEDD8 modification in viral proteins. Last, we reveal that MLN4924 has concentration-dependent biphasic effects on TPA- or SB-mediated lytic reactivation in PEL cells. These findings may potentially provide further insights into the regulation of KSHV lytic cycle activation and may have important implications for clinical trial design if MLN4924 is used in combination therapies.

Consistent with previous observations from Hughes et al. (33), we here showed that MLN4924 alone significantly induces the expression of the immediate early and early lytic proteins (ORF50, K8, and ORF45) in three PEL cell lines; however, only very low levels of K8.1 expression and viral particle release were produced from these MLN4924-treated cells (Fig. 1, 7, and 8). This uncoupling of action of MLN4924 in viral early lytic protein expression (including ORF50, K8, and ORF45) and in the progression of the later lytic events (K8.1 expression and virus release) may be due to the blockade of viral lytic DNA replication by MLN4924, as suggested by Hughes et al. (33). In order to elucidate the molecular mechanism by which MLN4924 promotes viral reactivation, we focused our studies on the transcriptional regulation of the ORF50 gene, a key viral immediate early gene (15, 16). Since MLN4924 inactivates CRLs, specific transcription factors that are known to modulate ORF50p transcription could be accumulated in PEL cells following MLN4924 treatment (Fig. 2). Not surprisingly, the levels of some tested transcription factors such as c-Jun and HIF-1α were elevated in PEL cells after exposure to MLN4924. However, transient reporter analysis revealed that the increased levels of c-Jun or HIF-1α caused by MLN4924 seemed not to be essential for ORF50p activation (Fig. 3 and 4). These results indicate that MLN4924 treatment may profoundly influence the activity of these tested transcription factors, probably due to improper posttranslational modifications of these transcription factors or dysregulation of other essential coactivator proteins in PEL cells. Although the levels of RBP-Jκ protein remained unchanged in PEL cells after treatment with MLN4924, the MLN4924-responsive element was mapped to RBP-Jκ-binding sites within the ORF50 promoter in PEL cells (Fig. 3 and 4). Intriguingly, we did not detect activation of the ORF50 promoter by MLN4924 in KSHV-negative cells, suggesting that viral factors may be required for MLN4924-mediated ORF50p activation.

Two viral transcription factors including ORF50 and LANA, which interact with RBP-Jκ (18, 25, 27), were subsequently included in our list of potential regulators involved in MLN4924-mediated activation of the ORF50 promoter. We ruled out the involvement of ORF50 in MLN4924-mediated ORF50p activation because MLN4924 could not increase autostimulation of the ORF50 promoter in KSHV-negative cell lines (data not shown). However, we did find that the ORF50p reporter construct could be reactivated by MLN4924 in KSHV-negative cells after cotransfection with a LANA expression plasmid (Fig. 5C and D). Mechanistically, we showed that MLN4924 acts to relieve the repressive effect of LANA on ORF50p transcription. Since MLN4924 treatment did not substantially affect the expression level of LANA in PEL cells or in 293T cells, the reduced activity of LANA in repressing the ORF50p transcription caused by MLN4924 could be due to alteration of LANA's posttranslational modifications or dysfunction of LANA-associated coregulators (5, 35). In the latter case, we did not find a significant change in levels of LANA-interacting transcription factors such as RBP-Jκ and KAP1 in PEL cells or in 293T cells after MLN4924 treatment (Fig. 2 and 5). In the former case, we did find that LANA is a neddylated protein in cells and could be deneddylated after treatment with MLN4924. These findings suggest that the neddylation status of LANA may be one of the key determinants controlling the assembly of the repressive complex on the ORF50 promoter. At present, several questions still need to be clarified to support the proposed hypothesis. First, it would be important to map the neddylation sites in LANA and make neddylation-deficient LANA mutants. The use of neddylation-deficient LANA mutants may help us clarify the role of LANA neddylation in the repression of the ORF50p transcription. Second, due to the fact that LANA is capable of interacting with cellular MDM2 (36, 37), an E3 ligase for ubiquitin and NEDD8 (38, 39), in infected cells, it would be interesting to investigate whether MDM2 can directly mediate neddylation of LANA. Third, in addition to ORF50p transcriptional regulation, it is also relevant to distinguish whether neddylation of LANA is associated with LANA's other regulatory functions, such as latent viral DNA replication and segregation in infected cells (7).

Emerging evidence has shown that MLN4924 is an anti-cancer drug effective against various types of human malignancies (28, 32). Due to the importance of the ubiquitin proteasome system in normal or cancer cells, a complete inactivation of CRLs by MLN4924 at high concentrations would severely influence cellular homeostatic balance and eventually induce cell death. However, partial inhibition of CRLs by MLN4924 at relatively low concentrations may potentially exert the opposite effects on cell growth in different cell types (40). As noted above, in cell proliferation assays we found that all three PEL cell lines treated with MLN4924 at low concentrations (0.1 and 0.3 μM) actually showed an increased growth rate at the early time points (1 or 2 days posttreatment) compared to the rate of untreated control cells (Fig. 8G to I). However, when these PEL cells were treated with higher concentrations (1 to 5 μM) of MLN4924, the cell viability of these treated cells was gradually reduced in a concentration-dependent manner (Fig. 8G to I). These results indicate that the different extents of protein deneddylation caused by MLN4924 may produce profound effects on multiple cellular activities. As with several anti-cancer drugs, MLN4924 is proposed to treat diseases or cancers in combination with other therapeutic agents (41, 42). Here, we unexpectedly found that MLN4924 possesses concentration-dependent biphasic actions in TPA- or SB-mediated viral lytic replication in PEL cells. Although the underlying regulatory mechanisms operated by MLN4924 in both TPA-treated and SB-treated PEL cells could be different, we consistently found that low concentrations (0.1 to 0.3 μM) of MLN4924 have a promoting effect on TPA- or SB-mediated viral reactivation in PEL cells, whereas high concentrations (>1.0 μM) of MLN4924 significantly inhibit the completion of TPA- or SB-mediated viral reactivation. According to our findings, it is important that different concentrations of MLN4924 in combination with other chemical or biological stimuli (or other therapeutic agents) may potentially produce very distinct effects on the progression of the KSHV lytic program. We therefore propose that, in clinical use, the dosage selection of MLN4924 in combination therapy should be cautious regarding the treatment of patients who are positive for KSHV.

## MATERIALS AND METHODS

### Cell cultures, reagents, and transfections.

All PEL cell lines, including BCBL1 (8), BCP1 (43), and BC3 (44), were grown in RPMI 1640 medium supplemented with 15% fetal bovine serum (FBS). 293T cells (45) were cultured in high-glucose Dulbecco's modified Eagle's medium (DMEM) supplemented with 10% FBS. 293T(BAC16) cells with the KSHV genome carried on BAC16 (34) were maintained in DMEM containing 10% FBS and hygromycin at 200 μg/ml. BJAB, a KSHV-negative B cell lymphoma cell line (46), was maintained in RPMI 1640 medium with 10% FBS. For viral lytic induction, BCBL1 and BCP1 cells were treated with TPA (30 ng/ml), while BC3 cells were treated with sodium butyrate (3 mM) or TPA (30 ng/ml). All PEL cell lines were routinely verified based on their cell morphology, the stable maintenance of the KSHV genome, and their expression profiling of viral lytic genes during KSHV lytic cycle induction. Additionally, under the normal condition, cell cultures were tested for mycoplasma contamination at regular intervals (every 6 months) by PCR (Venor GeM mycoplasma PCR detection kit; Minerva Biolabs) in the laboratory. MLN4924 was obtained from Active Biochem (A-1139; Maplewood, NJ). Transfection experiments were performed using Lipofectamine 2000 according to the manufacturer's instructions (Invitrogen).

### Western blot analysis.

Western blot analysis was carried out as described previously (47). Briefly, protein samples were resolved on an 8% to 12% polyacrylamide gel and then transferred onto a polyvinylidene difluoride (PVDF) membrane (Bio-Rad) where the target protein was probed with a specific antibody. All Western blot analyses done in the study were performed using at least two independent sets of cell lysates, and similar results were obtained across all experiments. The anti-ORF50 antibody was generated as described previously (48). Antibodies to FLAG (A8592; Sigma), K8 (sc-57889; Santa Cruz), ORF45 (sc-53883; Santa Cruz), K8.1 (sc-65446; Santa Cruz), LANA (13-210-100; Advanced Biotechnologies), RBP-Jκ (sc-271128; Santa Cruz), KAP1 (ab-22553; Abcam), Nrf2 (sc-722; Santa Cruz), HIF-1α (610958; BD Biosciences), Fos (sc-52; Santa Cruz), Jun (sc-1694; Santa Cruz), phospho-Jun (13-2100; Advanced Bioteck, Inc.), SP1 (CS200631; Millipore), hemagglutinin (901501; BioLegend), NEDD8 (PA5-17476; Thermo Fisher), cullin 1 (sc-11384; Santa Cruz), PARP (9532; Cell Signaling), cleaved caspase-3 (9664; Cell Signaling), and actin (sc-47778; Santa Cruz) were purchased commercially.

### Plasmid construction.

The pORF50p(−3801/+10)/luc reporter plasmid that contains the ORF50 promoter from −3801 to +10 was described previously (47). To construct ORF50 promoter deletion mutants shown in Fig. 3A, the indicated regions of the ORF50 gene promoter were amplified by PCR and cloned into pGL3-Basic (Promega). To further define the MLN4924 response elements, single or triple copies of specific transcription factor-binding motifs were cloned into pE4luc (49), a reporter plasmid containing a minimal adenovirus E4 promoter. The LANA expression vector was constructed by inserting a PCR-amplified LANA-coding gene fragment into pFLAG-CMV-2 (Sigma) at EcoRI and XbaI sites. The PCR primers used for amplification of the LANA-coding gene fragment were 5′-TTTGAATTCGATGGCGCCCCCGGGAATGCGCCT and 5′-AGGTCTAGAGGTGTGGCTTTTATGTCATTTCCT. The pCMV-HA-NEDD8 expression vector was obtained from OriGene (Rockville, MD).

### Luciferase reporter assay.

Cells (7 × 10^5^) were transfected with a fixed amount (0.8 μg) of plasmid DNA. The reporter assays were performed according to the manufacturer's protocol for the luciferase reporter assay system (Promega). Fold activation was calculated as luciferase activity in the presence of MLN4924 divided by that in the absence of MLN4924. The value of fold activation represents at least three independent experiments, with duplicate samples in each transfection.

### Analysis of LANA neddylation.

293T or 293T(BAC16) cells that were transfected with the plasmids indicated in Fig. 6A and B were cultured in medium with or without MLN4924. In similar experiments, BCBL1 and BJAB cells were also left untreated or treated with MLN4924. At 24 h posttreatment, cells were harvested and lysed in 300 μl of the lysis buffer, a solution obtained by mixing buffer I (5% SDS, 150 mM Tris-HCl [pH 6.7], 30% glycerol) and buffer II (25 mM Tris-HCl [pH 8.2], 50 mM NaCl, 0.5% NP-40, 0.1% sodium azide, 0.1% SDS) in a ratio of 1:3 (50, 51). After sonication and incubation at 4°C for 10 min, supernatants were collected by centrifugation and diluted with 1,800 μl of phosphate-buffered saline containing 0.5% Nonidet P-40. Immunoprecipitation was performed by mixing protein lysates with anti-FLAG antibody (A8592; Sigma-Aldrich) or anti-LANA antibody (13-210-100; Advanced Biotechnologies) for 2 h at 4°C. The protein mixtures were then incubated with protein A/G-agarose beads (Upstate) for another 1.5 h at 4°C. Bound proteins were analyzed by immunoblotting.

### Quantification of viral particles.

Culture supernatants were collected from BCP1, BC3, or BCBL1 cells that were left untreated or treated with MLN4924 or other lysis-inducing agents for 3 days. To remove contaminating DNA, culture supernatants were treated with DNase I (30 units/ml) for 1 h. The reaction was stopped by EDTA (5 mM), and samples were further treated with 0.1% SDS and 100 μg/ml proteinase K at 37°C overnight. Viral DNA extraction and a subsequent quantitative TaqMan PCR were performed as described in our previous study (34).

### Cell proliferation assay.

Cell proliferation was assessed by XTT {2,3-bis (2-methoxy-4-nitro-5-sulfophenyl)-5-[(phenylamino) carbonyl]-2H-tetrazolium hydroxide} assay (Roche) according to the manufacturer's instructions. Briefly, absorbance of the converted dye was measured at a wavelength of 450 nm using a microtiter plate reader. A reference wavelength of 630 nm was used to assess nonspecific readings. Duplicate samples were analyzed for each cell line, and each experiment was repeated at least twice.
